# Suspected Ticagrelor-Induced Bullous Fixed Drug Eruption

**DOI:** 10.7759/cureus.13890

**Published:** 2021-03-15

**Authors:** Tiffany Kawall, Rajeev Seecheran, Valmiki Seecheran, Sangeeta Persad, Naveen A Seecheran

**Affiliations:** 1 Internal Medicine, Eric Williams Medical Sciences Complex, Champ Fleurs, TTO; 2 Internal Medicine, Eric Williams Medical Sciences Complex, Champs Fleurs, TTO; 3 Cardiology, Eric Williams Medical Sciences Complex, Champs Fleurs, TTO

**Keywords:** bullous, exanthema, fixed drug eruption, hypersensitivity, ticagrelor

## Abstract

We describe a case of a suspected cutaneous hypersensitivity reaction to ticagrelor. The patient displayed a localized bullous fixed drug eruption after being loaded with ticagrelor, which resolved with oral antihistamines and topical steroids after one week. Clopidogrel and rivaroxaban were successfully administered as alternative antithrombotic therapy without any apparent further hypersensitivity skin reaction.

## Introduction

The risk of thrombotic events is lower with dual antiplatelet therapy (DAPT) in acute coronary syndrome (ACS) patients who received percutaneous coronary intervention (PCI) [1]. Ticagrelor is an oral, reversible P2Y12 adenosine diphosphate (ADP) receptor blocker [2]. As per the European Society of Cardiology 2017 guidelines, DAPT with ticagrelor and aspirin is the preferred treatment in patients with ACS, irrespective of an invasive or noninvasive treatment strategy being adopted (IB level of evidence) [3]. Clopidogrel is an oral prodrug in which the active metabolite specifically and irreversibly inhibits the P2Y12 ADP receptor [4]. As a predecessor to ticagrelor, clopidogrel use was near-ubiquitous in ACS and once deemed the "second best-selling drug" in the world [5].

Hypersensitivity skin reactions to ticagrelor are exceedingly rare, with less than 40 cases reported by the United States Food and Drug Administration (FDA) [6]. The index case was described by Quinn et al. in 2014 [7]. Clopidogrel skin hypersensitivity reactions are well-documented and occur in approximately 1% of patients, of which over 90% present with a maculopapular rash [8]. Currently, there is a paucity of data with respect to cross-reactivity between ticagrelor, clopidogrel, and the emergence of hypersensitivity skin reactions despite having a similar mechanistic effect of ADP blockade.

We describe a case of localized bullous fixed drug eruption, which resolved after transitioning from ticagrelor to clopidogrel and rivaroxaban.

## Case presentation

A 44-year-old South Asian male with no significant medical or family history presented to the emergency department with typical chest pain and shortness of breath. He did not report any significant social history, and his review of systems was unremarkable. His vital signs indicated systolic blood pressures of 156/92 mmHg, heart rate of 108 beats per minute, respiratory rate of 20 breaths per minute, with an oxygen saturation of 97% on room air. The brief physical examination was normal. An emergency 12-lead electrocardiogram revealed marked, 6-millimeter ST-segment elevation in leads V1-V6 with reciprocal ST-depressions in the inferior leads, II, III, and aVF.

He was immediately administered oral aspirin, ticagrelor load, high-intensity rosuvastatin in addition to an intravenous nitroglycerin infusion and subsequently proceeded for primary PCI. During cardiac catheterization, it was discovered that the patient had the much dreaded "widow-maker" lesion - a proximal left anterior descending (LAD) artery occlusion with high-grade thrombus burden. He successfully underwent transfemoral drug-eluting stent (DES) implantation after thrombus aspiration with a Boston Scientific Corporation (Marlborough, Massachusetts) SYNERGY® everolimus DES.

Post-procedure, the patient developed translucent bullous lesions with surrounding erythema on the distal flexor aspect of the right forearm, for which he promptly received intravenous hydrocortisone and chlorphenamine. At this juncture, the tentative diagnosis was a contrast-media reaction (Ultravist®️ 370, Bayer AG, Leverkusen, Germany). During the subsequent hospitalization, the patient was hemodynamically stable. He was subsequently discharged on guideline-directed medical therapy on DAPT with ticagrelor, high-intensity statin, low-dose beta-blocker, and angiotensin-receptor neprilysin inhibitor, and mineralocorticoid receptor antagonist, in addition to oral antihistamine and topical steroid cream therapies. A skin biopsy was not performed, as there were no available dermatologists during the coronavirus disease 2019 (COVID-19) pandemic.

**Figure 1:**
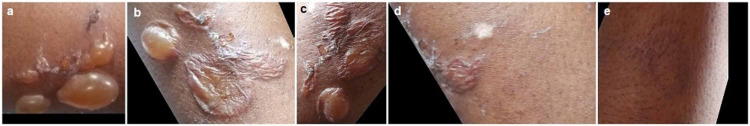
Chronological evolution of the suspected ticagrelor-induced cutaneous hypersensitivity syndrome a. Post-primary percutaneous intervention. Multiple tense bullous lesions appeared (within one hour) on the distal flexor aspect of the right forearm. b. Day 5. Some of the bullous lesions spontaneously ruptured with serous drainage. c. Day 7. Bullous lesions still persisting and re-appearing on ticagrelor therapy. Ticagrelor has been switched to clopidogrel and rivaroxaban. d. Day 14. Almost all lesions have ruptured and are healing by secondary intention on a modified antithrombotic regimen. e. Day 30. The lesions have all disappeared with post-inflammatory hyperpigmentation on the current regimen.

The patient contacted the interventional cardiologist one-week post-procedure and alerted him that the lesions were worsening despite the therapies. At that time, a measured decision was taken to transition him to clopidogrel (loading dose of 300 milligrams, followed by daily maintenance dose, 75 milligrams) and rivaroxaban 2.5 milligrams twice daily. Over the following four days, the bullous lesions spontaneously ruptured, with resolving erythema and induration. After a further three-day period, the lesions almost fully healed with areas of hyperpigmentation. There was no generalized body or mucosal involvement. Provocation tests with ticagrelor were not performed (Figure 1).

## Discussion

Oral antiplatelet cutaneous hypersensitivity reactions often present the clinical conundrum of drug discontinuation at the expense of possibly fatal cardiovascular complications such as stent thrombosis [9-10]. These cutaneous hypersensitivity reactions usually occur within one week of DAPT initiation. As aforementioned, it was previously attributed to a suspected contrast media reaction, and there was a concern of differential diagnoses of bullous Stevens-Johnson syndrome (SJS) and toxic epidermal necrolysis (TEN). However, the rash worsened during the first week while on ticagrelor twice-daily maintenance therapy, prompting its discontinuation and subsequently improved after transitioning to clopidogrel and rivaroxaban. The patient was reticent to retry ticagrelor as a provocative challenge and, unfortunately, dermatohistopathology, immunohistochemistry, and hematoxylin and eosin (H&E) staining to exclude an autoimmune condition were not obtained due to logistical reasons for the ongoing COVID-19 pandemic. The suspected ticagrelor-induced lesions were unique with respect to several issues. The bullous lesions appeared at the distal flexor aspect of the right forearm despite transfemoral access being utilized for the primary PCI. Despite generalized bullous fixed drug eruption being distributed throughout the body with absent to mild mucosal involvement, our patient only displayed right distal forearm involvement [11-14]. Additionally, there were no systemic or constitutional symptoms such as fever, malaise, weight loss, or arthralgias. Furthermore, these tense, bullous localized lesions have not yet been described with ticagrelor administration.

Hypersensitivity skin reactions to ticagrelor are relatively rare [6,10]. To the authors' knowledge, there are currently less than 40 reported hypersensitivity skin reactions with ticagrelor. In contrast, the incidence of skin rashes in the second and third-generation thienopyridines is 2.4% and 2.8%, respectively [11]. Like the thienopyridines and their generations, ticagrelor blocks the P2Y12 ADP receptors. However, ticagrelor is a structurally different cyclopentyl-triazolo-pyrimidine and has a different binding site, making it an allosteric antagonist [12]. Cross-reactive hypersensitivity might be less likely susceptible to thienopyridine antiplatelet drugs. Currently, the major cardiovascular societies have no formal definitive guidelines; however, the treatment options typically include drug discontinuation, desensitization therapy, transitioning to alternative antiplatelet therapies, and the administration of oral steroids in clinically high-risk patients [10]. In our patient, whom we deemed high risk due to his "widow-maker" lesion and high thrombus burden, we opted to transition to dual antiplatelet therapy with aspirin, clopidogrel, and low-dose direct oral anticoagulation with rivaroxaban. This regimen fit our particular clinical scenario and was associated with a reduction in-stent thrombosis and mortality [13]. The patient was also administered high potency topical steroids (augmented betamethasone dipropionate 0.05% twice daily) with oral antihistamines (desloratadine 5 milligrams once daily) with resolution within the ensuring week.

## Conclusions

We report a case of a suspected ticagrelor-induced bullous fixed drug eruption, which was conservatively managed with topical steroids and antihistamines. The physician should be aware of the rare dermatologic manifestations of ticagrelor, an antiplatelet therapy rapidly gaining traction in treatment algorithms for acute coronary syndromes.
